# Meta-Analysis of the Diagnostic Value of Cell-free DNA for Renal Cancer

**DOI:** 10.3389/fmolb.2021.683844

**Published:** 2021-08-11

**Authors:** Yipeng Xu, Yingjun Jiang, Mingke Yu, Jianmin Lou, Mei Song, Han Xu, Yingying Cui, Xiaowei Zeng, Qibo Wang, Hanyun Ma, Zongping Wang, Shaoxing Zhu, Guorong Li, An Zhao

**Affiliations:** ^1^ Department of Urology, Cancer Hospital of the University of Chinese Academy of Sciences, Zhejiang Cancer Hospital, Hangzho, China; ^2^ Hangzhou Traditional Chinese Medicine Hospital, Zhejiang Chinese Medical University, Hangzhou, China; ^3^ The Second Clinical Medical College, Zhejiang Chinese Medical University, Hangzhou, China; ^4^ Department of Ultrasound, Cancer Hospital of the University of Chinese Academy of Sciences, Hangzhou, China; ^5^ Central Research Laboratory, Children’s Hospital of Nanchang University, Hangzhou, China; ^6^ Translational Radiation Oncology Research Laboratory, Department of Radiooncology and Radiotherapy, Charite University Hospital, Berlin, Germany; ^7^ Comprehensive Cancer Center, Charité Universitätsmedizin, Berlin, Germany; ^8^ Department of Urology, North Hospital, University of Jean-Monnet, Saint-Etienne, France; ^9^ Experimental Research Center, Zhejiang Cancer Hospital, Institute of Cancer Research and Basic Medicine, Cancer Hospital of the University of Chinese Academy of Sciences, Hangzhou, China

**Keywords:** cf-DNA, renal cancer, diagnosis, liquid biopsy, meta-analysis

## Abstract

Cell-free DNA (cf-DNA) has been reported to represent a suitable material for liquid biopsy in the diagnosis and prognosis of various cancers. We performed a meta-analysis of published data to investigate the diagnostic value of cf-DNA for renal cancer (RCa). Systematic searches were conducted using Pubmed, Embase databases, Web of Science, Medline and Cochrane Library to identify relevant publications until the 31st March 2021. For all patients, we evaluated the true diagnostic value of cf-DNA by calculating the number of true positive, false positive, true negative, and false negative, diagnoses by extracting specificity and sensitivity data from the selected literature. In total, 8 studies, featuring 754 RCa patients, and 355 healthy controls, met our inclusion criteria. The overall diagnostic sensitivity and specificity for cf-DNA was 0.71 (95% confidence interval (CI), 0.55–0.83) and 0.79 (95% CI, 0.66–0.88), respectively. The pooled positive likelihood ratio and pooled negative likelihood ratio were 3.42 (95% CI, 2.04–5.72) and 0.36 (95% CI, 0.23–0.58), respectively. The area under the summary receiver operating characteristic curve was 0.82 (95% CI, 0.79–0.85), and the diagnostic odds ratio was 7.80 (95% CI, 4.40–13.85). Collectively, our data demonstrate that cf-DNA has high specificity and sensitivity for diagnosing RCa. Therefore, cf-DNA is a useful biomarker for the diagnosis of RCa.

## Introduction

Renal cancer (RCa) is the 13th most common cancer in the world and accounts for 2.4% of all cancers; the highest incidence of RCa has been reported in developed countries (Capitanio et al., 2019). More than 73,000 new cases of RCa are diagnosed in the United States every year, with 330,000 new cases globally (Capitanio et al., 2019; Siegel et al., 2019). Although developments in computed tomography and magnetic resonance imaging have increased the proportion of diagnoses for the early stages of RCa, only 47% of RCa patients with locoregional disease can survive more than 5 years (Choueiri and Motzer, 2017). Moreover, metastases occur in 4.2–7.1% of RCa patients when the diameter of the tumor is <4 cm, and this relates to an 8% 5-years survival rate for these patients (Lughezzani et al., 2009). To date, there is no biomarker for RCa like PSA for prostate cancer or EGFR for lung cancer. The discovery and application of novel biomarkers for RCa are still expected in clinical.

A number of recent publications have been reported that the potential for using cell-free DNA (cf-DNA) for the diagnosis of certain diseases (Jiang and Lo, 2016; Bianchi and Chiu, 2018). During tumorigenesis and the progression of cancer, it is likely that cf-DNA will be released into a patient’s blood by cells undergoing apoptosis, or by exosomes (Bardelli and Pantel, 2017). Consequently, cf-DNA, consisting of nucleic acid chains from various cell types, could be detected in the blood, stools, urine or saliva (Stewart et al., 2018). A variety of strategies could therefore be used to analyze cf-DNA, including real-time polymerase chain reaction (RT-PCR), digital PCR or next generation sequencing (van Ginkel et al., 2017; Cohen et al., 2021; Yang et al., 2021). cf-DNA has also been found to be able to serve as the prognostic indicator for tumor progression and drug resistance in cancer patients (Adalsteinsson et al., 2017; Remon et al., 2017; Alix-Panabières and Pantel, 2021). Since then, several studies assessing the value of cf-DNA in RCa have been published. However, the diagnostic performance of this novel biomarker has not been evaluated systematically. Therefore, the purpose of our study was to assess the diagnostic performance of cf-DNA for the detection of RCa.

## Materials and Methods

### Search Strategy

We carried out systematic literature searches to identify relevant publications in the PubMed, embase databases, Web of Science, Medline and Cochrane Library up to the March 31, 2021, without language or date restrictions.

The search strategy included the following terms: (“kidney neoplasms” OR “kidney cancer” OR “renal cancer”) AND (“diagnosis” OR “biomarker”) AND (“overall survival (OS)” OR “disease-free survival (DFS)” OR “progression-free survival (PFS)” OR “prognosis” OR “survival” AND “circulating tumor DNA” OR “cell-free nucleic acids” OR “ct-DNA” OR “cf-DNA”. Three researchers (Yipeng Xu, Yingjun Jiang and Mingke Yu) independently assessed the eligibility of each potentially relevant study by screening the titles and abstracts. Disagreements between the two researchers were resolved by discussion with two additional researchers (An Zhao and Shaoxing Zhu). Additional publications were identified by searching the reference lists of the selected papers.

### Inclusion Criteria

The inclusion criteria were as follows (Capitanio et al., 2019): at least one diagnostic or prognostic parameters for cf-DNA detection was reported in RCa patients, or could be calculated from the published data (Siegel et al., 2019); samples were collected from the peripheral blood (Choueiri and Motzer, 2017); the techniques were clearly stated in the articles; and Lughezzani et al. (2009) studies must feature negative controls.

### Exclusion Criteria

The exclusion criteria were as follows (Capitanio et al., 2019): repeated or overlapped publications which included the same study population and genes (Siegel et al., 2019); experiments based exclusively on cell lines or tumor tissue rather than clinical samples; and Choueiri and Motzer (2017) studies with a poor sample size (≤10).

### Data Extraction

All eligible studies were independently reviewed by two investigators (Y.J. Y.X.). The following items were extracted from each article: first author’s name, year of publication, number of patients, TNM stage, sample origin, methods of DNA detection, detection markers, and information relating the article’s quality. A range of diagnostic data were also extracted, including specificity, sensitivity, true positive (TP) rate, false positive (FP) rate, true negative (TN) rate, and false negative (FN) rate. We also acquired a range of survival data, including OS, PFS, hazard ratio (HR), *p* value, Kaplan-Meier survival curves, and 95% confidence intervals (95% CIs). Engauge Digitizer 4.1 was used to read the Kaplan-Meier curves in order to identify articles with accurate HRs.

### Risk of Bias in Individual Studies and Synthesis of the Results

Deek's funnel plot and Quality Assessment of Diagnostic Accuracy Studies (QUADAS) 2 tool were adopted to analyze qualitative publication bias, and a P-value of <0.05 was considered statistically significant. Risk-of-bias assessment was performed independently by two authors (Y.J. Y.X.) according to the Quality Assessment of Diagnostic Accuracy Studies (QUADAS) 2 tool (Whiting et al., 2011). Disagreement was solved by a third party (M.Y.). This tool provides a measure of the risk of bias and applicability over four domains of interest (Figure 2A). No publication bias in the pooled diagnostic effects was determined by Deek's funnel plot (p = 0.43) (Figure 2B).

### Statistical Methods

The systematic review and meta-analysis were performed using RevMan Version 5.3 and STATA 11.0 (Stata Crop). Diagnostic variables, including positive likelihood ratios (PLR), negative likelihood ratios (NLR), and summary receiver operating characteristic curves (SROC), were analyzed by STATA 11.0 (Stata Crop), and the diagnostic odds ratio (DOR) was analyzed by Meta-DiSc software, version 1.4. Specificity was defined as the proportion of patients with no cf-DNA detection in the blood samples when compared with all negative control volunteers without RCa. Sensitivity was defined as the proportion of RCa patients containing cf-DNA in their blood samples. TP, FP, TN, and FN, were calculated by analyzing the specificity, sensitivity, and the number of people enrolled in each group (experimental group and control group). Significant heterogeneity was defined as when *p* ＜ 0.05 or I^2^ ＞50%, and a random-effect model was used for heterogeneity analysis.

## Results

### Study Selection

**FIGURE 1:**
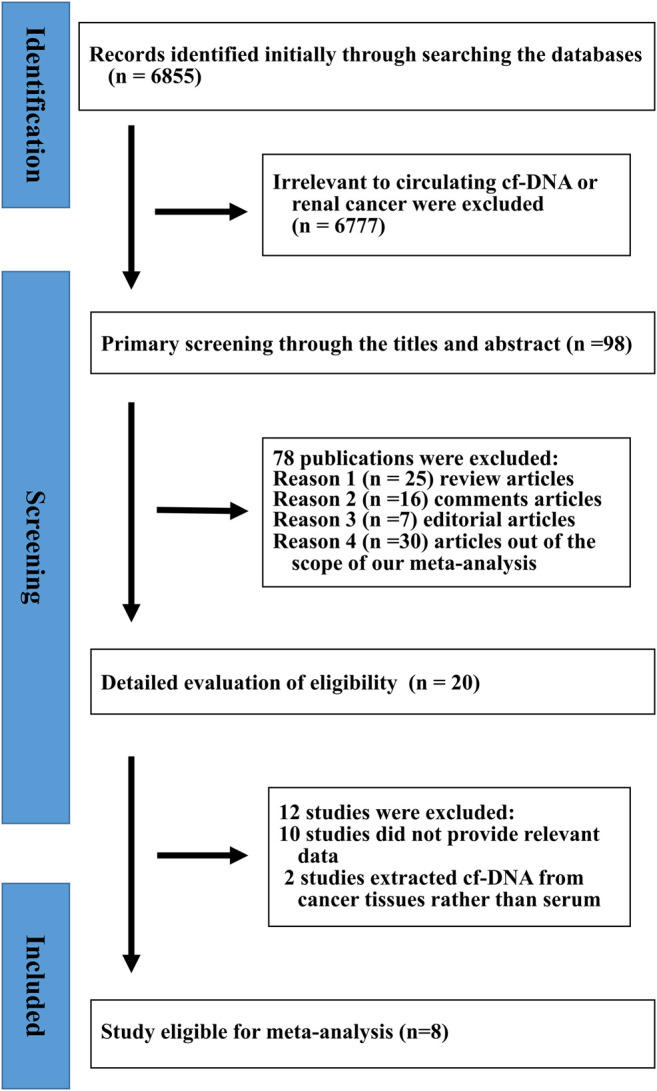
Flowchart describing the selection of publications for meta-analysis.

In total, we retrieved 6,855 articles. Of these, 6,777 articles were excluded because they did not specifically refer to cf-DNA and RCa. By reviewing each title and abstract, we identified 25 review articles, 16 comments, seven editorials, and 30 articles, that were outside of the scope of our meta-analysis. Twenty studies were recognized as potentially relevant publications, and a full-text review was performed to identify data relating to diagnoses and prognoses. As shown in Figure 1, careful screening and verification identified eight studies that were eligible for meta-analysis. The main characteristics and details of these eligible studies Salinas-Sanchez et al. (2021), Lasseter et al. (2020), Yamamoto et al. (2018), Lu et al. (2016), Wan et al. (2013), De Martino et al. (2012), Ellinger et al. (2012), Hauser et al. (2010) are summarized in Table 1. These eight eligible studies featured a total of 754 patients, with a median sample size of 87 (range: 33–229, mean: 94). In total, 355 controls were enrolled by the eight eligible studies, of which 312 were healthy individuals and 43 were patients with benign renal tumors (De Martino et al., 2012). Four studies included patients at stages I-IV, while the remaining three studies featured patients in stages I-III (De Martino et al., 2012; Ellinger et al., 2012; Wan et al., 2013). The number of TP, FP, FN, and TN, cases in these studies are shown in Table 1. One of the eligible articles were performed in Spain, two studies were performed in East Asia (China and Japan), three studies were performed in Germany, and the other was performed in the United States.

**TABLE 1:** Characteristics of Studies Evaluating the cf-DNA Levels of Patients with Renal Cancer

Study ID	Region/Year	Sample size (case/Control)	Mean age (case/Control)	Sample/Method	Cf-DNA characterize	TNM (i/II/III/IV)	TP	FP	FN	TN	Sensitivity/Specificity
Salinas-Sánchez et al (2021)	Spain/2021	82/20	59.7/59.5	Plasma cf-DNA/qPCR	Fragments of cf-DNA	58(I + II)/24(III + IV)	32	2	50	18	39.1%/90.0%
Lasseter et al (2020)	American/2020	34/34	NM	Plasma cf-DNA/cfMeDIP–seq	Methylation score of cf-DNA	20/3/6/9	34	4	0	30	100%/88%
Yamamoto et al (2018)	Japan/2018	92/41	68/57	Plasma cf-DNA/qPCR	Fragments of cf-DNA	58/4/15/15	58	9	34	32	63.0%/78.1%
Lu et al (2016)	Germany/2016	229/40	NM	Plasma mitochondrial cf-DNA/qPCR	Fragments of cf-DNA	108/19/94/2	160	5	69	35	70.0%/88.0%
Wan et al (2013)	China/2013	92/44	NM	Plasma cf-DNA/qPCR	Fragment of cf-DNA	59(I + II)/33/0	65	13	27	31	70.6%/71.2%
De Martino et al (2012)	American/2012	157/43	64.7/62.5	Serum cf-DNA/qPCR	Total cf-DNA	92(I + II)/65/0	80	3	77	40	51.0%/93.0%
Ellinger et al (2012)	Germany/2012	33/79	64.8/31.5	Serum mitochondrial cf-DNA/qPCR	Integrity of mitochondrial cf-DNA	21/1/11/0	27	45	6	34	81.8%/43.0%
Hauser et al (2010)	Germany/2010	35/54	66/28.5	Serum cf-DNA/qPCR	Integrity of cf-DNA	21/1/11/2	26	20	9	34	74.2%/62.9%

qPCR, Quantitative real-time PCR; NM, Not Mentioned; TP, True Positive; FP, False Positive; FN, False Negative; TN, True Negative.

### Detection of Cf-DNA

Cf-DNA was primarily detected by next generation sequencing or PCR-based method, and it could be characterized by composition (size, fragment or integrity), concentration (total, panel or specified gene) or genetic characteristics (methylation or nucleotide variants) (Table 1). Of these, three studies extracted DNA from serum Hauser et al. (2010), De Martino et al. (2012), Ellinger et al. (2012), while the other five studies extracted DNA from plasma (Wan et al., 2013; Lu et al., 2016; Yamamoto et al., 2018; Lasseter et al., 2020; Salinas-Sanchez et al., 2021). The blood volume required for the detection of cf-DNA varied from 0.8 to 3 ml. Notably, in the three enrolled studies, 1 ml of serum was used for cf-DNA detection (Hauser et al., 2010; De Martino et al., 2012; Ellinger et al., 2012). All of the studies collected blood samples prior to initial treatment.

### Risk of Bias Within Studies

**FIGURE 2:**
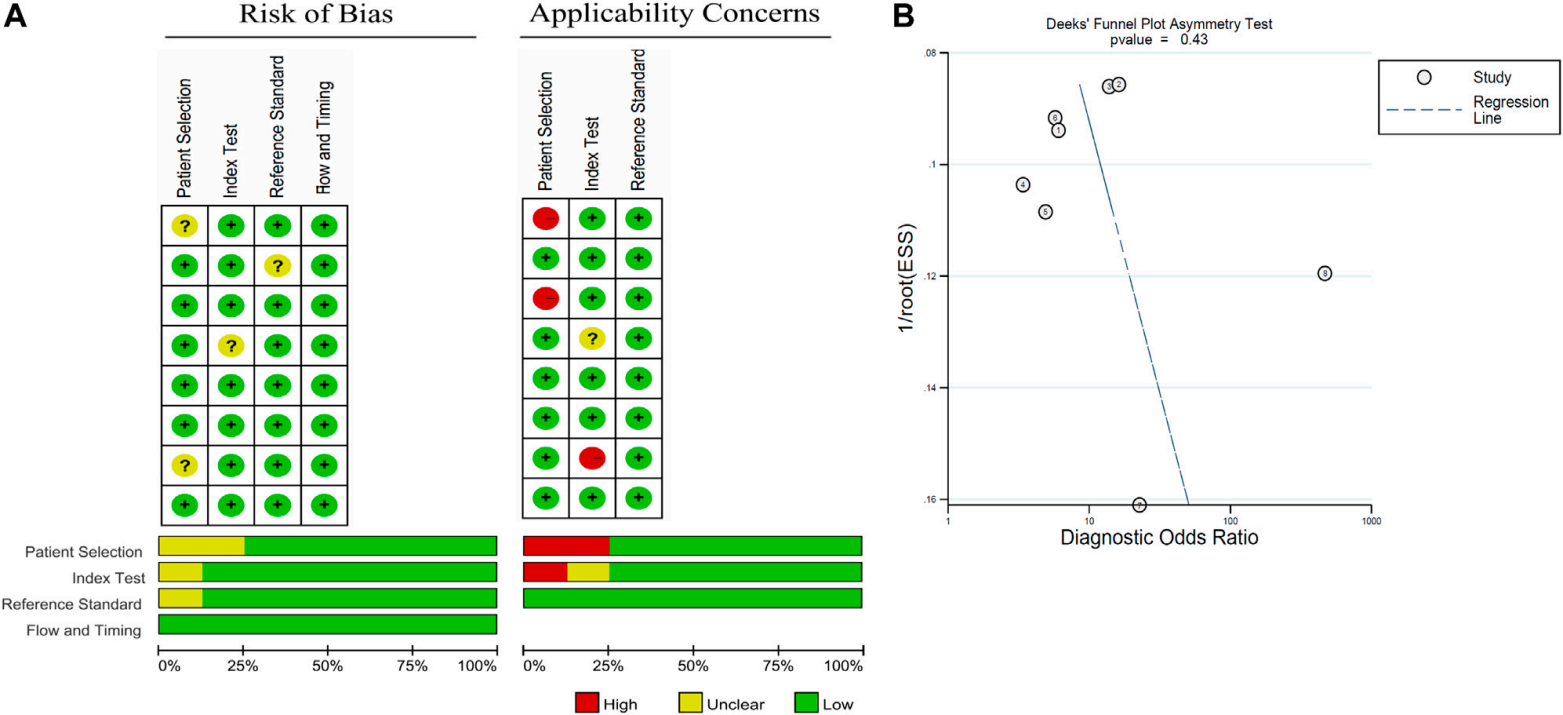
Graphical overview of the overall risk of bias and applicability judgements for the 8 studies included according to the Quality Assessment of Diagnostic Accuracy Studies (QUADAS) 2 tool and Deek's funnel plot.

The quality of the selected studies was evaluated in accordance with the QUADAS-2 criteria; the results of these evaluations are shown in Figure 2. Two studies were considered to be low-risk with regards to bias and applicability, and the other six studies were estimated as suboptimal for unclear risk in several areas, including patient selection, reference standards, and index testing. With DOR as the effect variable, the heterogeneity test gave a *p* value of 0.015, and an I^2^ value of 59.6%, suggesting that the heterogeneity was existed between these studies. In addition, meta-regression analysis was performed to analysis the heterogeneity. Among several potential variables, including the source of cf-DNA (serum or plasma), proportion of patients with RCa and region (Asia/USA/Europe), were not significant factors (*p* > 0.05, data not shown).

### Meta-Analysis of Diagnostic Value

**FIGURE 3:**
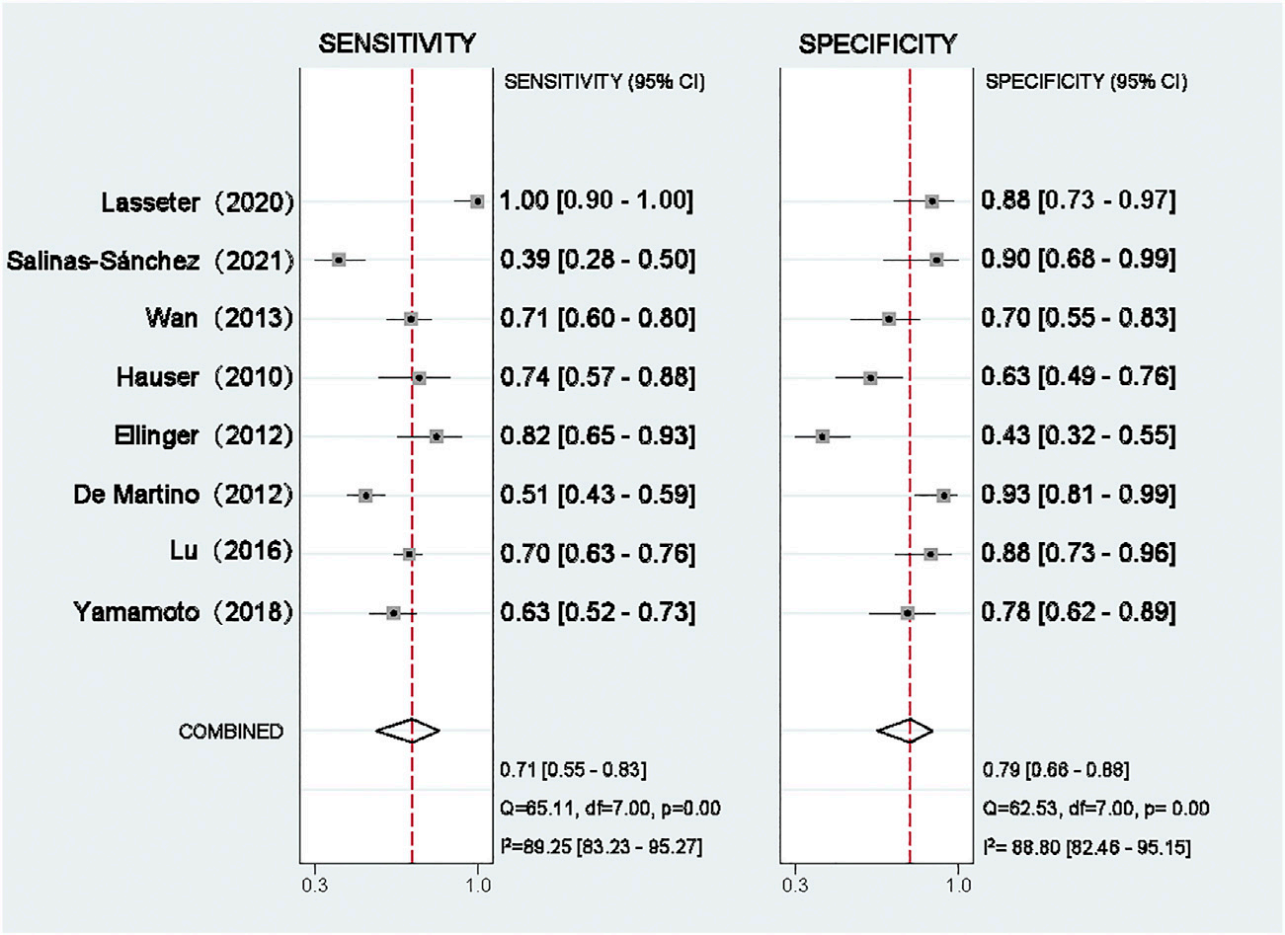
Forest plot of the pooled sensitivity and specificity.

All eight eligible studies were used to evaluate the diagnostic accuracy between cf-DNA expression and RCa. As shown in Figure 3, the overall diagnostic sensitivity and specificity were 0.71 (95% CI, 0.55–0.83) and 0.79 (95% CI, 0.66–0.88), respectively. The level of cf-DNA was significantly correlated with specificity (*p* < 0.001, I^2^ = 88.80%) and sensitivity (*p* < 0.001, I^2^ = 89.25%) (Figure 3).

**FIGURE 4:**
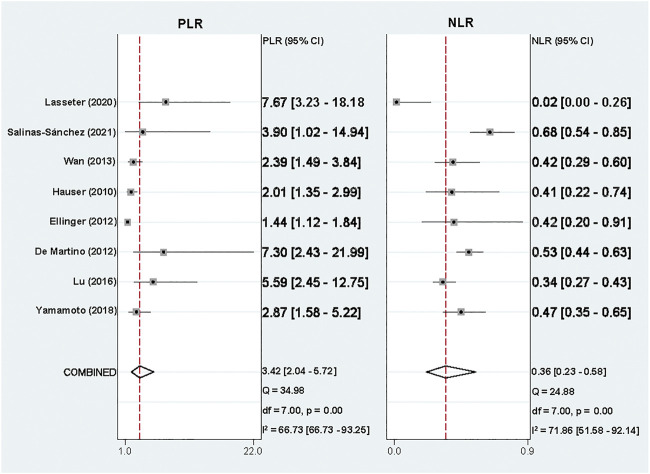
Forest plot of positive likelihood ratio and negative likelihood ratio.

**FIGURE 5:**
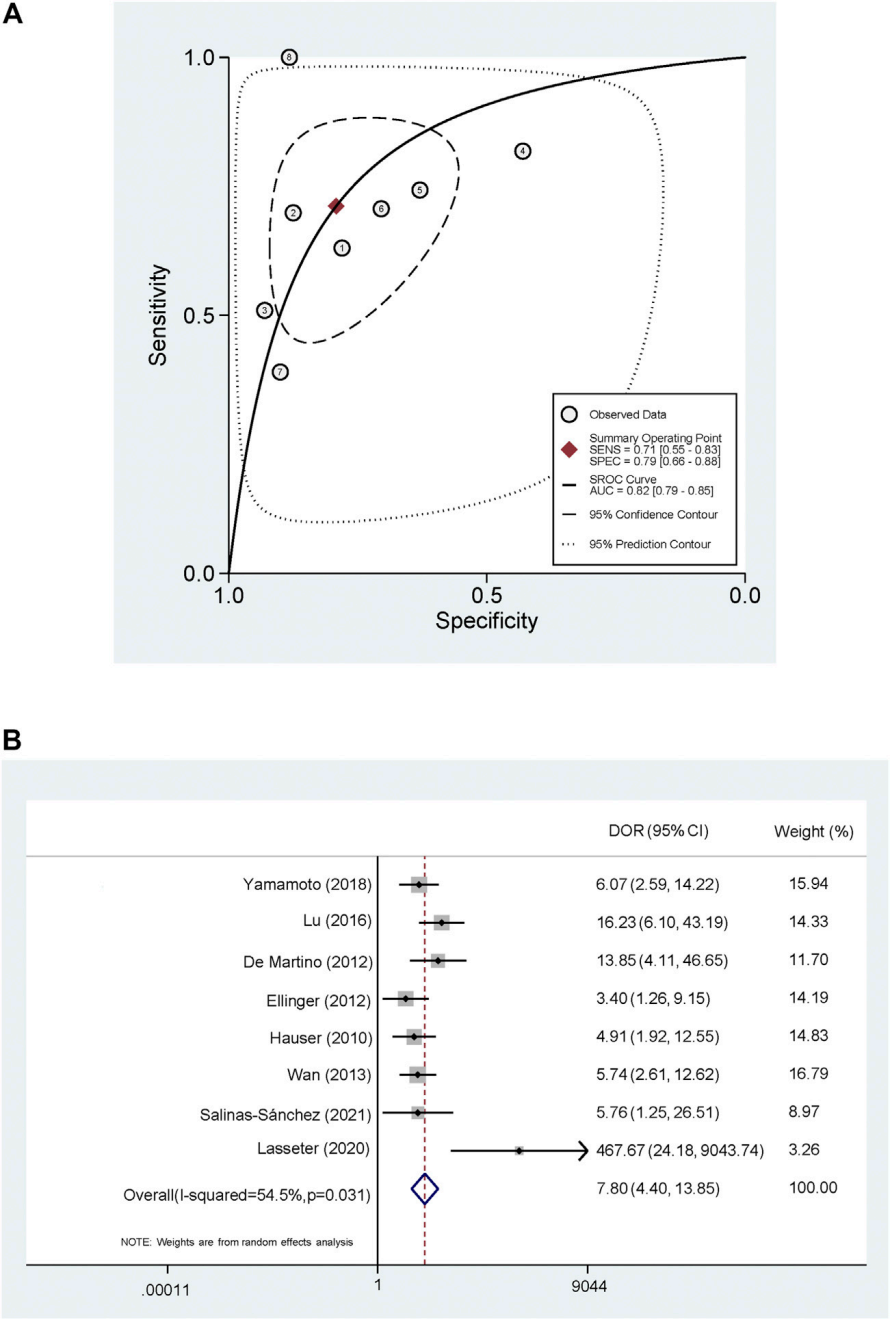
Summary receiver operating characteristic curve and Forest plot of diagnostic odds ratio **(A)** Summary receiver operating characteristic curve **(B)** Forest plot of diagnostic odds ratio.

The pooled PLR and NLR were 3.42 (95% CI, 2.04–5.72) and 0.36 (95% CI, 0.23–0.58) (Figure 4). The SROC was 0.82 (95% CI, 0.79–0.85) (Figure 5A) and the DOR was 7.80 (95% CI, 4.40–13.85) (Figure 5B).

### Prognoses

A total of six eligible studies De Martino et al. (2012), Wan et al. (2013), Lu et al. (2016), Yamamoto et al. (2018), Lasseter et al. (2020), Salinas-Sanchez et al. (2021) showed an association between cf-DNA and prognosis for patients with RCa. Two study investigated the association between cf-DNA expression and OS Lasseter et al. (2020), Salinas-Sanchez et al. (2021), while another study investigated the association between cf-DNA and DFS De Martino et al. (2012), these data could not be merged. As for the other three studies, one investigated the association between cf-DNA expression and PFS Yamamoto et al. (2018), while the other investigated the association between cf-DNA expression and RFS (recurrence free survival) (Wan et al., 2013; Lu et al., 2016); these data could not be merged. The specific details of these four studies are shown in Supplementary Table S1.

## Discussion

Early stage RCa is usually asymptomatic and is therefore usually discovered by chance (Ljungberg et al., 2019). The diagnosis of RCa still predominantly depends on radiological and histopathological examinations; however, these techniques are associated with exposure to radiation, and are in invasive. Unlike the case of prostate-specific antigen (PSA) for prostate cancer, there is no specific biomarker for diagnosing the early stages of RCa, or predicting disease progression in such patients. This reduces patient compliance and means that it is difficult for us to screen patients, and monitor patients over long periods of follow-up. The identification of a diagnostic indicator in peripheral blood samples of RCa patients, that could be used for diagnosis and screening, would be of significant clinical value.

Liquid biopsy is widely regarded as a new diagnostic technique for cancer (Husain and Velculescu, 2017). Recent research, involving patients with urogenital cancer Di Meo et al. (2017), indicates that circulating tumor cells, cell-free nucleic acids, circulating tumor DNA, circulating cell-free RNA, and extracellular vesicles and their cargo, extracted from blood and urine, have significant potential for monitoring disease status (Zhao et al., 2015; Li et al., 2017; Zhang et al., 2018). Compared with radiological and histopathological examinations, these new methods are minimally invasive and carry minimal risk, such methods may also provide us with the possibility to test patients continuously for disease recurrence and response to treatment.

The presence of fragmented DNA in the blood was first reported by Mandel and Metais Mandel (1948) in 1948. In recent decades, the detection of cf-DNA has been applied to various different types of cancer. For example, identifying the EGFR T790M mutation in plasma samples is already known to be an effective method for determining EGFR status in patients with non-small cell lung cancer (NSCLC) (Qiu et al., 2015). Furthermore, the loss of the EGFR T790M mutation in plasma is associated with early progression to advanced NSCLC patients receiving osimertinib (Cohen et al., 2021). The genomic profiles of ct-DNA have also been shown to closely match those of the corresponding tumors, with important implications for both molecular pathology and clinical oncology (Siravegna et al., 2017). Although the experience of cf-DNA detection in RCa patients is very limited, our ability to diagnose disease by liquid biopsy is highly likely to become increasingly powerful in the future.

To investigate the clinical utility of cf-DNA in the diagnosis of RCa, we performed a meta-analysis and found that the detection of cf-DNA conveyed an obvious advantage to the specificity of RCa diagnosis (specificity: 0.79; 95% CI: 0.66–0.88). Furthermore, the sensitivity of cf-DNA for the diagnosis of RCC was also high (sensitivity: 0.71; 95% CI: 0.55–0.83). In these analyses, higher PLR values indicated that the test results were more likely to be disease-related, while lower NLR values indicated that the test results were more likely to be disease-independent. The area under the ROC curve (AUC) was used to further evaluate the accuracy of these diagnostic tests, cf-DNA showed a high diagnostic ability (AUC = 0.82) to diagnose RCa (Figure 5). There are few studies on the prognosis of cf-DNA in RCa, it is not clear whether levels of cf-DNA expression could be used to evaluate patient prognosis. Interestingly, Yamamoto et al. (2018) have reported that patients with longer cf-DNA fragments (>160 bp) had a longer PFS than those with shorter fragments (<160 bp), suggesting that cf-DNA may have different release mechanisms between the normal cells and the tumor cells. In addition, multiple aspects can be assessed in circulating cf-DNA, including expression levels, integrity, methylation and mutations (Di Meo et al., 2017). As advances in laboratory technology and knowledge, the meta-analysis of diagnostic value of subtype of cf-DNA is needed in the future.

There are several limitations associated with our meta-analysis that should be taken into consideration. First, the lack of an appropriate cf-DNA gene target in RCa patients might contribute to the presence of bias. Like many other types of cancer, RCa is also considered as a malignancy with high histological and etiological heterogeneity. Further studies of specific target genes would help us to fully understand the use of cf-DNA detection for patients with RCa. Second, due to an enrichment of studies reporting positive results, it is impossible to exclude the possibility of selection bias. Other sources of bias may have arisen due to differences in detection equipment and materials, such as PCR primers and amplification systems.

## Conclusions

Based on the studies selected for meta-analysis, our data indicate that cf-DNA could serves as a liquid biopsy that is effective for the diagnosis of RCa.

## Data Availability

The datasets presented in this study can be found in online repositories. The names of the repository/repositories and accession number(s) can be found in the article/Supplementary Material.
