# Supplementary material for: Meta-Analysis of the Diagnostic Value of Cell-free DNA for Renal Cancer
Source: Front Mol Biosci. 2021 Aug 11;8:683844. doi: 10.3389/fmolb.2021.683844 (PMC8385273; doi:10.3389/fmolb.2021.683844)
Supplement: Supplementary file 1 [file Table1.DOCX]

**Table S1.** Summary of prognostic data of cf-DNA reported in the studies included in the current meta-analysis

| **Author** | **OS** | **DFS** | **PFS** | **RFS** |
| --- | --- | --- | --- | --- |
| **Salinas-Sánchez**^16^ | √ | × | × | × |
| **Lasseter**^17^ | √ | × | × | × |
| **Yamamoto**^18^ | × | × | √ | × |
| **Lu**^19^ | × | × | × | √ |
| **Wan**^20^ | × | × | × | √ |
| **De Martino**^21^ | × | √ | × | × |

OS: overall survival; DFS: disease-free survival; PFS: progression-free survival; RFS: relapse-free survival.
